# Evaluating the dose, indication and agreement with guidelines of antimicrobial use in companion animal practice with natural language processing

**DOI:** 10.1093/jacamr/dlab194

**Published:** 2022-02-09

**Authors:** Brian Hur, Laura Y. Hardefeldt, Karin M. Verspoor, Timothy Baldwin, James R. Gilkerson

**Affiliations:** 1 Asia-Pacific Centre for Animal Health, Melbourne Veterinary School, University of Melbourne, Parkville, Victoria, Australia; 2 School of Computing and Information Systems, University of Melbourne, Parkville, Victoria, Australia; 3 School of Computing Technologies, RMIT University, Melbourne, Victoria, Australia

## Abstract

**Background:**

As antimicrobial prescribers, veterinarians contribute to the emergence of MDR pathogens. Antimicrobial stewardship programmes are an effective means of reducing the rate of development of antimicrobial resistance. A key component of antimicrobial stewardship programmes is selecting an appropriate antimicrobial agent for the presenting complaint and using an appropriate dose rate for an appropriate duration.

**Objectives:**

To describe antimicrobial usage, including dose, for common indications for antimicrobial use in companion animal practice.

**Methods:**

Natural language processing (NLP) techniques were applied to extract and analyse clinical records.

**Results:**

A total of 343 668 records for dogs and 109 719 records for cats administered systemic antimicrobials from 1 January 2013 to 31 December 2017 were extracted from the database. The NLP algorithms extracted dose, duration of therapy and diagnosis completely for 133 046 (39%) of the records for dogs and 40 841 records for cats (37%). The remaining records were missing one or more of these elements in the clinical data. The most common reason for antimicrobial administration was skin disorders (*n* = 66 198, 25%) and traumatic injuries (*n* = 15 932, 19%) in dogs and cats, respectively. Dose was consistent with guideline recommendations in 73% of cases where complete clinical data were available.

**Conclusions:**

Automated extraction using NLP methods is a powerful tool to evaluate large datasets and to enable veterinarians to describe the reasons that antimicrobials are administered. However, this can only be determined when the data presented in the clinical record are complete, which was not the case in most instances in this dataset. Most importantly, the dose administered varied and was often not consistent with guideline recommendations.

## Introduction

Antimicrobial resistance (AMR) is an emergent global health crisis that was estimated to be responsible for the loss of over 700 000 lives in 2016, and this figure is estimated to grow to over 50 million by 2050 without intervention.^1^ AMR reduces the therapeutic efficacy of antimicrobial treatment in both human and veterinary medicine with significant cost to patient health. As companion animals are able to acquire and exchange MDR pathogens with humans, and many of the same antimicrobial agents are used in human and veterinary medicine, companion animals can serve as a reservoir of AMR for in-contact people.^2–5^ This highlights the importance of appropriate antimicrobial usage in companion animal practice and increases the imperative to adopt strategies to mitigate AMR in small animal veterinary clinics.

Antimicrobial stewardship programmes are a way to reduce AMR in hospital environments.^6–10^ Measuring the effectiveness of antimicrobial stewardship programmes requires an understanding of the clinical indication for antimicrobial usage, as well as data on the antimicrobial usage patterns before and after the intervention. The clinical indication for antimicrobial use, the selection of an appropriate antimicrobial agent for that indication and administration of the correct dose for an appropriate duration of therapy are all key components of an appropriate use strategy that underpins good antimicrobial stewardship. The increasing number of practices with electronic health records, combined with data repositories such as VetCompass,^11^ Small Animal Veterinary Surveillance Network (SAVSNET),^12^ and Veterinary Medical Database (VMDB)^13^ provide the opportunity to systematically collect these data. However, the data are primarily in the form of free text and not readily queryable, making data retrieval and subsequent analysis difficult. Previous studies have evaluated frequency and types of antimicrobial usage patterns in the UK^14,15^ and Australia.^16,17^ Studies describing the reason for antimicrobial administration have relied on the indication recorded when an appointment was booked,^18^ or on the reason submitted in a claim to the pet insurance company.^19^ However, neither of these studies examined the reason for the antimicrobial use directly from the veterinarian’s clinical notes.

Extracting these data out of the clinical notes enables analysis of antimicrobial usage patterns directly based on the findings as recorded during the exam, and without interfering with the clinical workflow of the veterinarian. To perform such extraction and analysis at scale, we turn to automated methods based on natural language processing (NLP), which is a field of study that sits at the intersection of artificial intelligence and linguistics, with a broad goal of automating language analysis.^20^ In our context of veterinary notes, NLP can be used to overcome the challenges of manual labelling of such data, enabling large-scale extraction of key antimicrobial usage information in a structured format, to allow subsequent analysis.^21^ We focus on the extraction of actionable information from text,^22^ and specifically on the use NLP for text mining, which is the discovery of non-trivial knowledge from unstructured text.^23^

Models known as pre-trained contextualized language models have become popular in NLP, as they create a more nuanced and context-dependent representation of text than prior approaches.^24–27^ These models capture the latent syntax and semantics of text, and support the training of task-specific models—such as identifying the reason for antimicrobial administration—more effectively with fewer labelled instances. Recent work in this area has integrated representations based on generalized texts, such as Wikipedia, with specialized texts, such as clinical notes, in order to more effectively analyse medical records.^28^ One such model, ‘VetBERT’,^17^ has been developed specifically for the veterinary domain was pre-trained using data from VetCompass Australia and further trained to classify the indication for which an antimicrobial was given out of free-text clinical notes.

The aim of this study was to characterize the reasons antimicrobials are given in veterinary practice, as described in the clinical notes by a consulting veterinarian. To this end, we apply VetBERT to the VetCompass Australia database. We also aim to determine details of the antimicrobial use, such as dose and length of administration, and assess the completeness of the electronic medical records to explore whether antimicrobial administration agrees with guidelines.

## Materials and methods

De-identified clinical data from 137 companion animal practices was sourced from VetCompass Australia (Version 0.3) for the period 1 January 2013 to 31 December 2017 inclusive.^29^ The antimicrobial agents administered and dosage information in each consultation were identified from free-text notes and collated using pre-existing NLP methods.^16,30,31^ Where a unit is defined as a single capsule, tablet or millilitre of the antimicrobial, the dose extracted consists of the unit size, unit dose and frequency of administration. For example, *one half 50 mg tablet given twice daily* would be calculated as 50 mg for unit size, 0.5 for the dose unit and 2/day for the frequency. The patient weight and the total antimicrobial units dispensed were extracted from structured fields in the VetCompass record. The total daily dose of a medication was calculated by (unit size × dose unit × daily dose frequency)/(weight of patient). The length of administration was calculated from (total number of units dispensed)/(total daily units). Where the item was injectable, the length of administration was fixed to one for the purposes of this report. A sample size of 97 is required to be 95% confident that estimated accuracy is within 10% of the actual range, based on Cochran’s formula for the representativeness of proportions.^32^ The accuracy of the automated dosage calculations created from the extracted data were assessed by randomly selecting 100 of the cases and manually calculating dosage.

A model referred to as VetBERT was used to classify the indication for disease.^17^ VetBERT was created using a model known as ClinicalBERT^28^ as a base and then using additional pretraining steps as described by Devlin *et al*.^24^ using the entire corpus of 15 million clinical notes from VetCompass Australia to provide a good representation of the veterinary clinical text. One of 38 possible indications for antimicrobial use was obtained using VetBERT for each record (Table S1, available as Supplementary data at JAC-AMR Online). Further steps were taken to evaluate the performance of the model on records from our specific dataset. First, 50 records were randomly selected and labelled by three veterinarians to determine the inter annotator agreement of the veterinarians. Second, 100 records labelled by the model were labelled by a single veterinarian to determine the accuracy of the labels created by VetBERT.’ Third, a further 400 records labelled by the model as ‘no indication recorded’ were randomly selected and split up, and each record was reviewed by one of three veterinarians to confirm the absence of a discernible indication.

Code was written in Python, and machine learning and statistical tests on algorithms performed with scikit-learn and TensorFlow libraries. All descriptive statistics, computations and visualizations were performed using Tableau 2020.^33^ Doses of antimicrobials were grouped by rounding to the nearest integer and given a tolerance of 10% before being compared with the Australian Veterinary Prescribing Guidelines from The University of Melbourne.^34^ Statistical significance was tested using Pearson’s χ^2^ test with *P* < 0.001 used to indicate significance.

## Results

Clinical data relating to 4  402 147 consultation records from 137 companion animal practices were aggregated for analysis (Table 1). Clinical records from 3  269 160 consultations for 513 962 dogs and 1 132 987 consultations for 199 358 cats were included in the analysis. Occasionally, a dog and cat were recorded within the same consultation. Of the consultation records analysed, 199 358 (26%) were cats and 513 962 (74%) dogs. Systemic antimicrobials were administered or dispensed in 109 719 (9.7%) of the cat consultations and 343 668 (11%) of dog consultations.

**Table 1.** Clinical data recorded in consultations when systemic antimicrobials were administered

Number of consultations	Dogs, *n* (%)	Cats, *n* (%)
Consultations with systemic antimicrobial events	343 668 (100)	109 719 (100)
Indication present	292 376 (85)	93 483 (85)
Weight present	204 233 (59)	65 997 (60)
Prescription labels present	291 320 (85)	92 356 (84)
Duration calculated	320 652 (93)	101 803 (93)
Dose calculated	143 878 (42)	43 996 (40)
Dose, duration and indication present	133 046 (39)	40 841 (37)

The manual dosage calculations exactly matched the calculations from the extracted data in 94% of cases. The Fleiss Kappa agreement score between the veterinarians was excellent (0.77) in determining the reason for an antimicrobial administration. The model had an accuracy of 82% in identifying the indication for an antimicrobial if one was dispensed, and accuracy of 81% when the model labelled ‘no indication recorded’. Of the records that were labelled with ‘no indication’ 80% were confirmed to have no identifiable indication.

**Figure 1.**
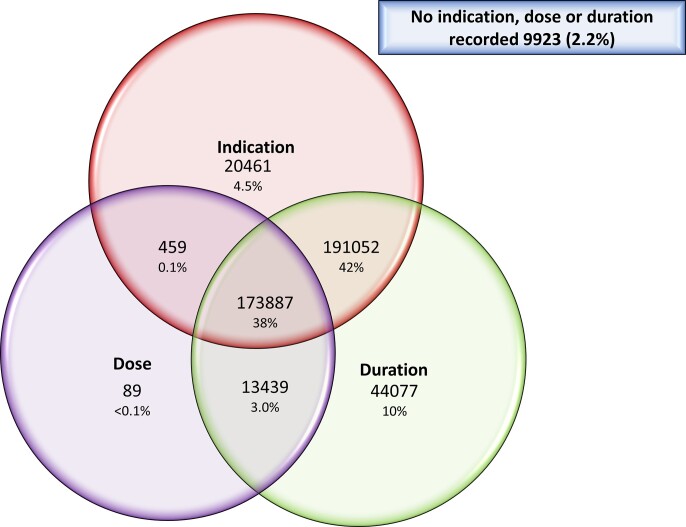
Number of consultation records where indication, dose and duration of antimicrobial use were extracted.

For all consultations where systemic antimicrobials were administered, dispensed or prescribed the dose, duration and indication for antimicrobial usage were extracted. Of the 453 387 consultations involving dogs and cats when antimicrobials were administered, only 173 887 (38%) recorded the indication, dose and duration (Figure 1). The remaining consultation records were lacking one or more of these variables, with 67 528 (15%) containing no discernible indication, 265 513 (59%) recording no dose rate, 30 932 (7%) with no recorded duration of treatment and 9923 (2.2%) of consultation records containing no indication, dose or duration data that could be extracted by the algorithms (Figure 1).

### Antimicrobial use in dogs

**Figure 2.**
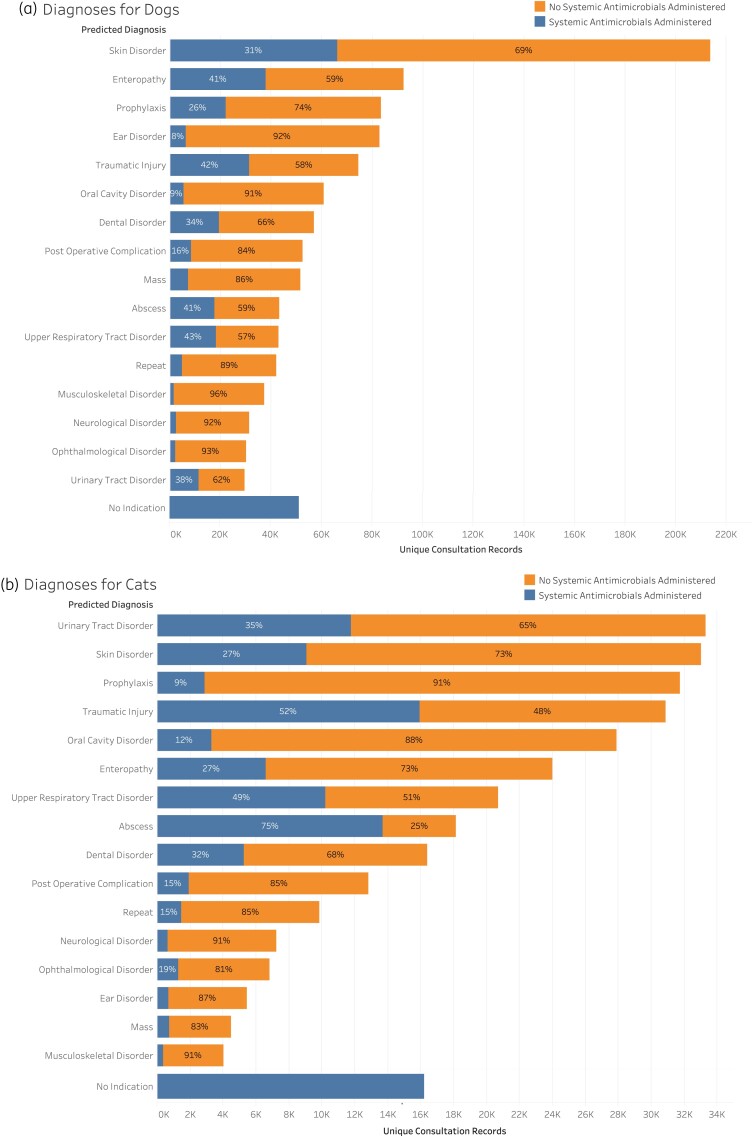
Proportion of consultations where systemic antimicrobials were used for common disease syndromes in dogs (a) and cats (b).

**Figure 3.**
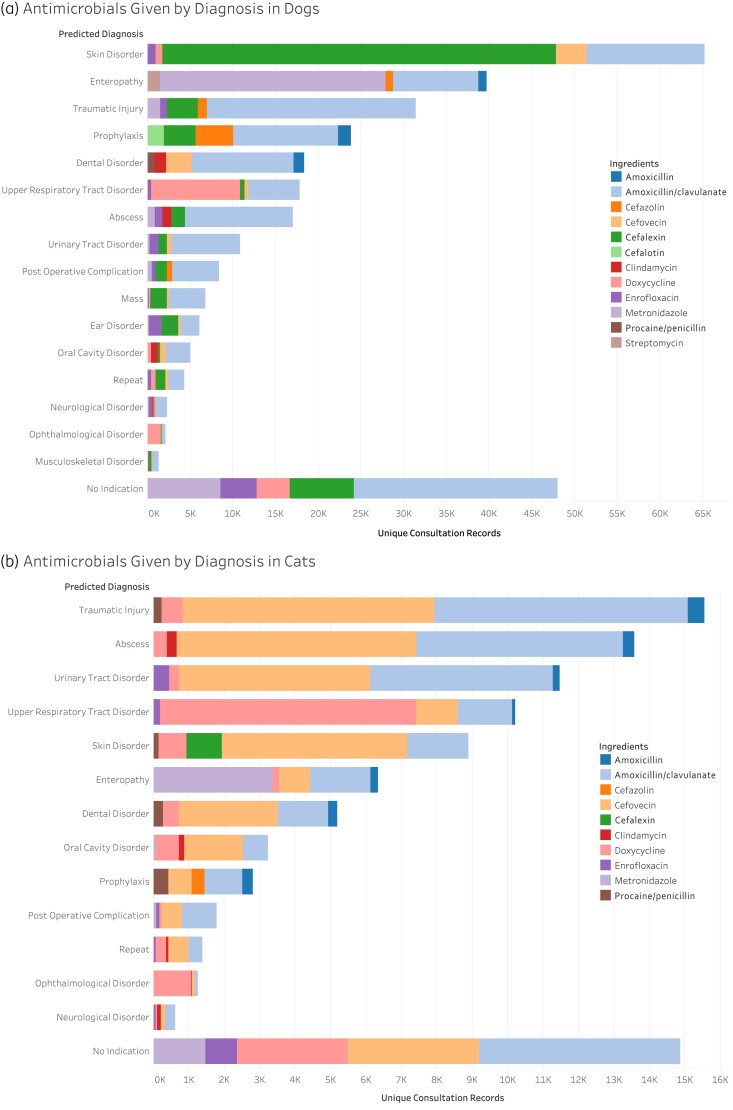
Five most frequently administered systemic antimicrobial agents used for common disease syndromes in dogs (a) and cats (b).

Systemic antimicrobials were most commonly administered to dogs in association with skin disorders (*n* = 66 198, 19% of total antimicrobials), enteropathies, (*n* = 37 746, 11% of total antimicrobials), and traumatic injuries (*n* = 31 146, 9% of total antimicrobials) (Figure 2a). Importantly, in 51 292 consultation records (15%) the clinical indication for systemic antimicrobial administration to dogs was not recorded (Figure 2a). The most common systemic antimicrobials prescribed for skin disorders in dogs were cefalexin (*n* = 46 091, 70%), amoxicillin/clavulanate (*n* = 13 738, 21%) and cefovecin (*n* = 3616, 5.5%) (Figure 3a), which is consistent with guidelines that recommend first-generation cephalosporins or amoxicillin/clavulanate. Disorders for which systemic antimicrobials were most commonly prescribed included enteropathies (37 746 of 92 266 records, 41%), traumatic injuries (31 146 of 74 470 records, 42%), abscesses (17 688 of 43 252 records, 41%) and upper respiratory tract disorders (18 251 of 42 794 records, 43%) (Figure 2a). Metronidazole was the most commonly administered antimicrobial agent for enteropathies (26 446 of 37 746 records, 70%), doxycycline was the most common systemic agent for upper respiratory tract disorders (10 369 of 18 251 records, 57%), while amoxicillin/clavulanate was the most commonly used antimicrobial for abscesses (12 647 of 17 688 records, 72%) and traumatic injuries (24 341 of 31 146 records, 78%) (Figure 3a).

### Antimicrobial use in cats

Systemic antimicrobials were most commonly administered to cats in association with traumatic injury (*n* = 16 924, 15% of total antimicrobials), abscesses (*n* = 13 804, 13% of total antimicrobials) and skin disorders (*n* = 12 107, 11% of total antimicrobials) (Figure 2b). The indication for antimicrobial administration was not recorded in 23 846 records (22%) (Figure 2b). The most common reason for systemic antimicrobial administration was traumatic injury in which 15 932 of 30 861 records (52%) were treated with systemic antimicrobials (Figure 3a).The most common systemic antimicrobials prescribed for traumatic injuries in cats include amoxicillin/clavulanate (*n* = 7150, 45%), cefovecin (*n* = 7105, 45%) and doxycycline (*n* = 601, 4%) (Figure 3b). Disorders for which systemic antimicrobials were most commonly prescribed included abscesses (13 699 of 18 148 records, 75%), traumatic injury (15 932 of 30 861 records, 52%) and upper respiratory tract disorders (10 196 of 20 689 records, 49%) (Figure 2b). Cefovecin was the most common antimicrobial given for abscesses (6776 of 13 699 records, 49%) where guidelines recommend no antimicrobials, or amoxicillin or ampicillin when animals are systemically unwell. Doxycycline was the most commonly used antimicrobial for upper respiratory tract disorders (7241 of 10 196 records, 71%) consistent with guideline recommendations.^34^

### Guideline comparison

**Figure 4.**
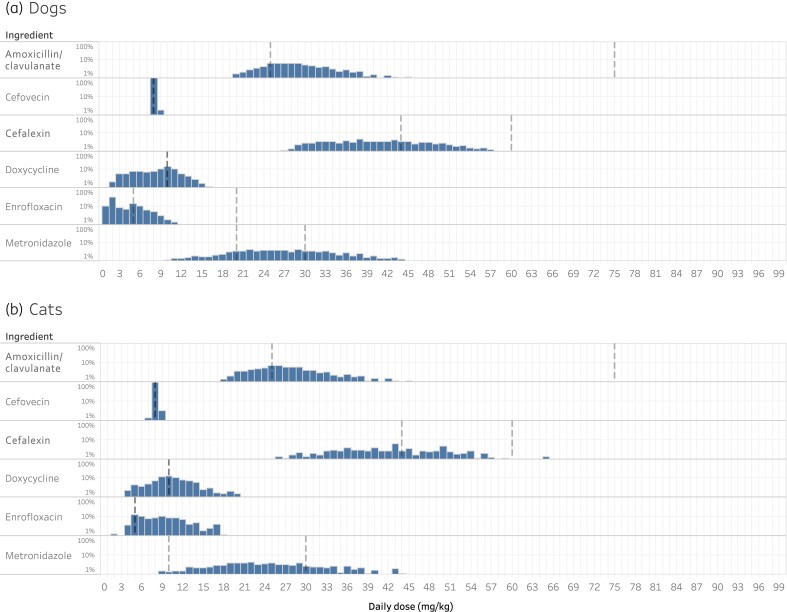
Dosage rate (mg/kg) extracted from records for commonly used antimicrobials in dogs (a) and cats (b). Recommended dosage, or dosage range, is indicated by the dotted lines (to the nearest mg/kg).

The daily doses of active ingredient (mg/kg) were calculated from the clinical records for the most commonly used antimicrobial agents, amoxicillin/clavulanate, cefalexin, enrofloxacin, metronidazole and cefovecin, for both cats and dogs (Figure 4). The reported daily dose in the consultation record for these medications was compared with the dose range recommended in the Australian Veterinary Prescribing Guidelines. The daily dose of administration for cefovecin had the highest proportion of doses within the guidelines, which was 95% for dogs (Table 2) and 93% for cats (Table 3). Doxycycline had the lowest proportion of administered doses that were compliant with the guidelines for dogs (35%) (Table 2), and enrofloxacin the lowest for cats (25%) (Table 3).

**Table 2.** Proportion of antimicrobial dispensing using the recommended dose for dogs

Ingredient	Overdosing	Dose within range (± 10%)	Underdosing
Amoxicillin/clavulanate	0.9%	88%	11%
Cefovecin	2.6%	95%	1.9%
Cefalexin	4.7%	53%	42%
Doxycycline	20%	35%	45%
Enrofloxacin	0.4%	47%	52%
Metronidazole	30%	56%	15%

**Table 3.** Proportion of antimicrobial dispensing using the recommended dose for cats

Ingredient	Overdosing	Dose within range (± 10%)	Underdosing
Amoxicillin/clavulanate	2.3%	80%	18%
Cefovecin	4.6%	93%	2.5%
Cefalexin	10%	56%	34%
Doxycycline	42%	34%	23%
Enrofloxacin	63%	25%	12%
Metronidazole	29%	69%	1.6%

## Discussion

In this study, we applied NLP techniques to evaluate a large dataset of veterinary notes to determine the reason for, and agreement with the guidelines of, antimicrobial use in dogs and cats. Veterinary studies evaluating agreement with the guidelines of antimicrobial use have been lacking from the literature until now, but similar studies have been performed in medical general practices and in urban hospitals.^35,36^ Evaluating agreement with guidelines in Australian hospitals has been fundamental in driving antimicrobial stewardship interventions.^37^ General medical practices are similar to veterinary practices in that they are often run by a single practitioner and cannot have dedicated staff to perform antimicrobial stewardship activities. The previous studies evaluating general medical practices used *post hoc* analysis, which used a field to specify the reason for the antimicrobial being dispensed. However, it was found that over 85% of these labels did not have the reason recorded, and it is likely the clinical notes themselves need to be examined to determine this.^38^ NLP methods, such as the ones demonstrated in our study, could potentially benefit these programmes by retrieving this information from the clinical notes themselves. Similarly, having an indication for an antimicrobial being dispensed could simplify the extraction of the indication of use. Further research is required to evaluate the utility of NLP techniques for investigating appropriateness in general medical practice.

The most common indication for antimicrobial administration in dogs was skin disorders and in cats it was traumatic injuries. This was similar to the findings from a study evaluating pet insurance claims in Australia^19^ and a study evaluating patients in the UK.^14^

Despite being the most common reason for antimicrobial administration, only 31% of the consultation records for dogs with a skin disorder were treated with systemic antimicrobials (Figure 2a). This would appear to be consistent with guidelines, which only recommend systemic antimicrobials when there are large areas of the body involved. The choice of amoxicillin/clavulanate for traumatic injuries in cats and dogs also appears to be in concordance with guidelines, although amoxicillin alone is recommended for abscesses and traumatic injuries rather than amoxicillin/clavulanate.^34^ However, further research is required to understand the clinical severity of these conditions and confirm the appropriateness of the chosen therapy.

While there are limitations of this study, it is a broad assessment of the antimicrobial selection for disease conditions and the dose of therapy chosen by the practitioner. Antimicrobial agent selection was broadly concordant with guideline recommendations in most common scenarios with some exceptions, such as traumatic injuries in cats. However, dose rate varied widely and may potentially be inappropriate (27%) when compared with the Australian Veterinary Prescribing Guidelines (Table 2). Underdosing of antimicrobials results in failure to achieve adequate MIC, which promotes AMR.^39^ This may be associated with a lower than expected number of records where the patient weight was recorded (60%), but may also be associated with the widely variable weight of the patients (Pomeranian versus Great Dane) and the lack of appropriately formulated dosages of common antimicrobials. A portion of the incorrect dosing is likely due to discrepancies between the label dose and available best-practice recommendations, as has been previously described in Australia.^40^ For example, while the available literature supports the dose rate of doxycycline being 5 mg/kg given twice daily,^41^ at least one label for doxycycline lists the dosage rate to be only 2.5 mg/kg for a daily dose.^42^ Similarly, at least one cefalexin label recommends 15 mg/kg every 12 h,^43^ where studies suggest that anything below 25 mg/kg should be given every 6–8 h to achieve the MIC necessary to be effective.^44^ Also, when no range is provided by guidelines, this can impact the proportion of cases where the appropriate dose is used. For example, doxycycline has a daily dose of 10 mg/kg; however, the combination of animal weight and tablet size may make it difficult to achieve a precise dose in many cases. Research is needed to investigate these factors.

Natural language processing is a powerful tool for automated data extraction and large-scale monitoring of antimicrobial administration on a large population level without the need for manual record labelling, as done in previous studies.^11,18,45^ However, these methods are limited by the completeness of the information recorded in the clinical notes. In addition, evaluating appropriate antimicrobial use requires the clinical record to clearly state the indication, the agent selected and the dose and duration of use. In this study, only 38% of the records analysed had all the information necessary to determine whether the antimicrobial usage was appropriate. The NLP algorithms were accurate, with a 94% accuracy for the dosing calculations correct and 80% accuracy in identifying the indication of the antimicrobial. The primary components of data missing included the weight of the animal, unit measurements for the antimicrobial agent, prescription details or a clear reason for the antimicrobial administration being described. While there is likely an overestimate of the doses missing from the records, the information necessary to perform this calculation, using the prescription or the concentration of the medication given, was not easily available. Missing data in medical records has also been reported in human health; a study investigating human primary care visits reported at least 14% of records were missing information that could adversely affect the clinical interpretation of the record.^46^ Further research is required to understand the reason for the missing data in many of the records, as it is not clear whether the data are not being entered at the time of the consultation, are entered in the wrong place or were lost during export of the records from the practices. The importance of having a complete clinical record is highlighted by the proportion of cats that received cefovecin, a highly critically important third-generation cephalosporin, when no indication for treatment was recorded. While giving a long-acting antimicrobial such as cefovecin may be appropriate, the reason for administration should be clearly indicated.

The analysis in this study was limited to VetCompass 0.3, and the methods had the same limitations previously described.^29^ The diseases were extracted at the syndrome level and specific clinical details of the syndromes were not extracted in enough detail to make an evaluation of appropriateness. This would require a detailed exploration of each disease syndrome and understanding what information is possible to extract from the clinical record. Additionally, the indication for the disease was counted on a per consultation record basis, and a patient seen multiple times and hospitalized may have multiple records for the same indication. While there were repeat visits labelled (11% in dogs and 15% in cats), these visits were not associated with the condition for which it was a repeat appointment and relied on text indicating a repeat visit. So, if there are two consultation records for the same patient with an abscess, and only one appointment with an antimicrobial administered, this could potentially be counted as one consultation for a patient that had an abscess and received an antimicrobial, and one consultation where a patient had an abscess and did not receive an antimicrobial. Further research would be required to understand the impact on each condition. This also made it unreasonable to evaluate the appropriateness of the duration in this study as prescription repeats and chronic conditions (i.e. skin conditions in dogs), with multiple consultations spread out over time, require additional methods to accurately assess duration of therapy.

There was also a lack of culture and susceptibility testing results in the data analysed. Increased use of bacterial cultures and susceptibility testing is likely to improve the clinical outcomes while reducing the importance ratings of the antimicrobials used. While there were labels with culture and sensitivity annotated in the original corpus used to train the disease syndrome classifier,^17,47^ there were not enough of these labels to train the model. Evaluation of the antimicrobial susceptibility of isolates obtained from canine urine cultures in Australia demonstrated that antimicrobial agents of lower critical importance could be selected without compromising efficacy,^48^ but further work is required to incorporate clinical pathology data into VetCompass and evaluate this in a large scale dataset.

## Conclusions

Utilizing natural language processing and records from VetCompass Australia, we have performed a large-scale analysis of the indication and dose of antimicrobial use in companion animal practice in Australia. We have demonstrated the utility of automated methods to support understanding why and how antimicrobials are being administered and describe how they agree with antimicrobial guidelines in instances where the data are able to be extracted. Further research is required to understand other factors relating to these behaviours and details of outcomes of treatments.

## Supplementary Material

dlab194_Supplementary_Data

## Supplementary data

Table S1 is available as Supplementary data at *JAC-AMR* Online.
