# Supplementary material for: Evaluating the dose, indication and agreement with guidelines of antimicrobial use in companion animal practice with natural language processing
Source: JAC Antimicrob Resist. 2022 Feb 9;4(1):dlab194. doi: 10.1093/jacamr/dlab194 (PMC8827557; doi:10.1093/jacamr/dlab194)
Supplement: dlab194_Supplementary_Data [file dlab194_supplementary_data.docx]

**Supplementary data**

**Table S1**. Labels used for indication of antimicrobial administration.

| Abscess |
| --- |
| Adverse Reaction to Drug |
| Appetite Disorder |
| Blood Test |
| Claw/Nail disorder |
| Dental Disorder |
| Disorder not Diagnosed |
| Ear Disorder |
| Endocrine System Disorder |
| Enteropathy |
| Female Reproductive Disorder |
| Fever (undiagnosed) |
| Foreign Body |
| Intoxication |
| Kidney Disorder |
| Lethargy |
| Liver Disorder |
| Lower Respiratory Tract Disorder |
| Male Reproductive Disorder |
| Mass |
| Musculoskeletal Disorder |
| Neoplasia |
| Neurological Disorder |
| Ophthalmological Disorder |
| Oral Cavity Disorder |
| Other Species |
| Palliative |
| Pancreatic Disorder |
| Post Operative Complication |
| Prophylaxis |
| Repeat |
| Skin Disorder |
| Traumatic Injury |
| Not recorded |
| Upper Respiratory Tract Disorder |
| Urinary Tract Disorder |
